# Consultant psychiatrists’ perspectives on occupational stress in child and adolescent mental health services (CAMHS)

**DOI:** 10.1007/s11845-021-02648-6

**Published:** 2021-07-27

**Authors:** Niamh Doody, Cliodhna O’Connor, Fiona McNicholas

**Affiliations:** 1 School of Medicine and Medical Science, University College Dublin, Dublin, Ireland; 2 Lucena Clinic, St John of God, Dublin, Ireland; 3 Department of Psychology, University College Dublin, Dublin, Ireland; 4 Children’s Health Ireland (CHI) At Crumlin, Dublin, Ireland

**Keywords:** Burnout, Child and adolescent mental health services (CAMHS), COVID-19, Occupational stress, Public perceptions, Qualitative

## Abstract

**Background:**

Occupational stress is increasingly recognised as key factor contributing to service quality, safety, and worker wellbeing, with clinician providers most at high risk.

**Objectives:**

To explore work-related stressors among consultant child and adolescent psychiatrists working in CAMHS.

**Methods:**

Fifty-two consultants completed an online questionnaire with free-text entries describing factors contributing to occupational stress in CAMHS in Ireland.

**Results:**

Content analysis indicated that consultants’ perception of working conditions revolved around six factors: organisational factors, human resources, adequacy of services, professional relationships, socio-political factors, and public perception. Both adequate skilled staff and funding, identified by 54% and 34% of respondents, were viewed as essential factors associated with occupational wellbeing, the most often cited concern (raised by 56% consultants) which contributed to occupational stress was of widespread public misunderstanding of CAMHS’ remit.

**Conclusions:**

Given decades of under-resourcing, ensuring adequate levels and expertise of staffing in the post-COVID-19 era must become a reality. However, less obvious and equally important is that of correcting any public misperceptions regarding CAMHS “core” business to ensure that available scarce resources are utilised most effectively, and that staff stress levels are minimised. To achieve this, active engagement between service users, providers and planners must be undertaken.

## Introduction

Occupational stress (OS) has been recognised as an essential quality metric in assessing and promoting work performance and satisfaction. Typical stressors at work leading to OS include excess work demand and inadequate resources, but increasingly, the workers’ internal experience of strain and lack of agency is becoming prominent [1]. Even when work demands are met, OS can occur when workers feel they are not supported or valued by their employers, colleagues, or those they provide a service for [2]. If unaddressed or unrecognised, burnout (BO), a term used to describe long-term, unresolved, work-related stress, with feelings of physical and emotional exhaustion, depersonalisation or cynicism, and a reduced sense of personal accomplishment may follow [3]. In Ireland, it is reported that 18% of the workforce experience work-related stress, with rates accelerating faster than in other EU countries [3].

Whilst many hypothetical models have been developed to examine occupational stress, the demand-control-support model is the most widely applied [4]. This model proposes that high workload (demand), accompanied by low levels of work-related decision authority and little collegial or employer support, is a toxic breeding ground for OS and BO. In Ireland, medical services are led by consultants who assume clinical responsibility for all patients. Consultants’ ability to influence staff and resource allocations varies and necessarily impacts decision-making latitude. Furthermore, in most mental health services, demand has increased and outstrips availability, as evidenced by long waiting lists. Child and adolescent mental health services (CAMHS) are particularly vulnerable to these risks, with referrals increasing by 24% between 2012 and 2018; long waitlists; and many benefits operating at lower than 50% of recommended levels [5]. The current national mental health policy, “sharing the vision” has acknowledged ongoing resource difficulties within CAMHS [6].

Professionals working in clinical settings have higher occupational stress rates than those in other groups [3], and elevated stress levels can negatively impact the quality of care patients receive [7]. A survey of Irish hospital doctors reported high burnout rates in one out of three doctors, with rates exceeding international norms [8]. As a group, consultant psychiatrists are noted to be exposed to and expected to manage stress from multiple sources, making them a particularly vulnerable group [9]. Furthermore, it has been demonstrated that consultant child and adolescent psychiatrists in Ireland report high levels of work-related and personal BO [10]. Of those surveyed, only 21% felt valued in their job, and 69% said that they had seriously considered changing jobs; both factors positively associated with higher rates of BO.

## Objectives

This study’s objective was to explore the personal perspective of consultant child and adolescent psychiatrists working in CAMHS and identify factors contributing to their level of occupational stress (OS), offering an opportunity to inform service development and provision.

## Methods

A survey was designed using the SurveyMonkey platform and a website link sent by the College of Psychiatry, Ireland (CPI) to all consultant child and adolescent psychiatrists registered with the Irish Medical Council and CPI members (n = 112). Participants were invited to complete the questionnaire, which took approximately 10–15 min. Two reminder follow-up emails were sent at three monthly intervals (May–August 2017) to maximise the data collection period. All responses were anonymised. The study’s main outcome measure was the Copenhagen Burnout Inventory (CBI), and results have been reported (McNicholas et al., 2020). Open-ended questions included the following: (i) what would make a difference to the quality of the CAMHS services offered, (ii) what would improve the public perception of CAMHS, and (iii) to add any comments they wished relating to CAMHS funding, quality, challenges, opportunities or services in Ireland.

## Participants

Fifty-two consultant child and adolescent psychiatrists replied (46% response rate). The majority (*n* = 42, 81%) worked in CAMHS outpatient community settings, with a smaller number (*n* = 7, 13%) working exclusively in an inpatient setting (five in a specialist CAMHS inpatient unit and two in a paediatric hospital) (Table 1). Three did not indicate their work environment. Respondents (*n* = 51, 98%) were, in general, seasoned clinicians with on average 14.5 years as a consultant (range 1–30 years). To protect the anonymity of the respondents, the exact location and names of services were not recorded.

**Table 1:** Professional and service characteristics

Total sample	n = 52, 46% response rate
Work place setting: n = 49	CAMHS OPD; *n* = 42, 81% CAMHS inpatient: *n* = 7, 13%
Clinical years’ experience: n = 52	Mean 14.5 years, median = 13.0, SD = 6.233, range 1–30 years
What percentage of your week do you think you spend on what you consider to be outside your area of responsibility or 'not core' responsibility of CAMHS? n = 52	Mean = 3.8, median = 4 (20–30%), SD = 3.83

*CAMHS* child and adolescent mental health services, *OPD* outpatients’ department or community setting

“n” represents the numbers who answered each question

## Analysis

A content analysis as described by Elo and Kyngäs [11] was performed using ATLAS.ti. This method was chosen over thematic analysis as it allows for a closeness to data and provides an opportunity for quantification of data [12]. Efforts to ensure trustworthiness were considered at each stage of design and analysis, ensuring the appropriateness of participants and providing adequate sample description, carefully outlining the organisation and interpretation of data and presentation of analysis codes. Researchers’ bias was considered and managed by analysis initially conducted by a non-CAMHS consultant (COC) and then reviewed and discussed with a consultant (FMcN) to minimise risk of bias in data interpretation.

The specific components of the analysis were as follows: responses to the three free-text questions were read through several times and recurring ideas were noted. This informed the development of a coding frame that captured the patterns present in the data. The coding frame was applied to the data, with each response “tagged” with codes reflecting its content. An individual response could have multiple codes assigned if multiple ideas were present.

The coding frame contained 32 codes, which were organised into six superordinate categories. There were the following: organisational factors, human resources, adequacy of services, professional relationships, socio-political factors, and public perception (Appendix I).

## Results

### Improving the quality of CAMHS services

**Fig. 1:**
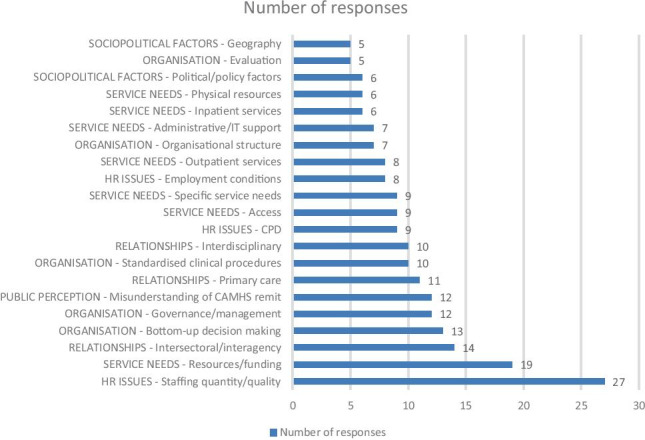
Factors that may improve the quality of CAMHS services

Fifty consultant child and adolescent psychiatrists responded to the free text linked to this question. Each response was assigned at least one code. Issues raised by more than five respondents are presented in Fig. 1.

The factor perceived to be most essential and endorsed by 27 participants related to team staffing, both numbers and degree of expertise. Quality improvements were considered to follow should “management hear our views on staffing”. Respondents requested “appropriate involvement of CAMHS consultants in decisions, including staffing, being made about their teams”.

Nine respondents called for more resourcing of training and CPD opportunities including “a training budget”.

Eight additionally recommended improvements in employment conditions to support the recruitment, retention, and performance of staff. “There is an urgent need to address the recruitment crisis in psychiatry and other sectors like nursing”. The second most common response (n = 19) was a generic call for more resources or funding and for service leads to be involved, with respondents urging planners “to listen to the clinicians who work in CAMHS in terms of needs & to give appropriate levels of funding to meet service needs”.

Thirteen respondents called for more involvement of clinical staff in the management decisions that affect service delivery. Twelve responses included a critical comment about current governance or management and the unrealistic pressures imposed on under-resourced teams. One respondent requested that management should be mindful of service limitations and “stop harassing consultants …” and “stop pressuring clinical staff to take on operational duties”, allowing them to continue with their clinical responsibilities. Another respondent perceived manager’s to be inadequately informed of CAMHS’ role, responsibility, and practice and suggested that the recruitment of “managers who actually understand what CAMHS is would be a start”. Another recognised the benefit of having “operational managers (embedded) in service provision and ensure weekly collaboration with consultant”.

Seven suggested specific changes to organisational structures, such as the composition of clinical teams or services’ locations. Specific reconfigurations included a proposal to create “child psychiatry clinical directors and a separate CAMHS CHO management teams with turnover of leads after 3 years to maintain performance”.

Consultants perceived that team members needed to be appointed; with the necessary skill set, their performance needed to be regularly reviewed by line managers, and “underperforming staff needs to be identified,” and roles and responsibilities were clearly defined in job plans. Despite consultants being “responsible for the patient care”, respondents felt that they “have no say in their team staffing or composition or line management”. Many respondents highlighted the need for greater skill set and flexibility in their teams; “Efforts should be made to employ more experienced staff in the multidisciplinary team, and more effort should be given to recruiting a specific skill base for specific posts”; and staff should be willing to deal the CAMHS “core business” and “must adopt a more pragmatic approach to case work”. One consultant proposed a more medical model to CAMHS and suggested to “disband MDTs leaving psychiatry and nursing” to provide the clinical service. Others emphasised the need for staff to be flexible and be “willing to work with acute emergency psychiatry” instead of others who “refuse to do so”.

Ten respondents noted the need to implement standardised clinical procedures for referral and therapeutic pathways and day-to-day running of service. Nine mentioned problems regarding access, with some specific proposals or recommendations for comprehensive on-call services, “24-h access to CAMHS either in community or emergency departments”, and that there should “be both equality of access and consistency across all CAMH services”. Improvements to outpatient and inpatient services were recommended by 8 and 6 respondents, respectively. Seven called for improvements to administrative and/or IT support, 6 for physical resources such as office space, and five for greater evaluation or auditing of service provisions.

The need for better coordination with other aspects of the health, social care, or education was mentioned by 14 respondents, focusing on TUSLA, with a request to “tackle the poor standards of assessment”. Consultants felt there needed to be “clarity about the role and of relationship between other agencies” to stem “the repeated attempts to derogate responsibility”. Eleven respondents mentioned the need for an increased role and remit in delivering mental health services by primary care. Twelve consultants stated that CAMHS services were compromised by “misunderstanding of CAMHS” role and remit. Ten noted the importance of effective interdisciplinary relationships.

### Improving public perception of CAMHS

**Fig. 2:**
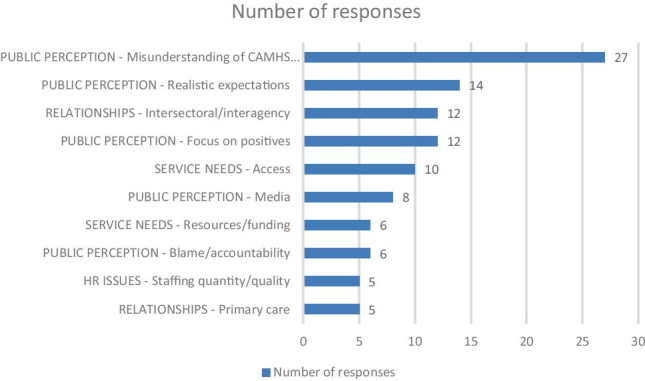
Improving public perception of CAMHS

There were 48 responses to this question—issues mentioned by more than five people displayed in Fig. 2.

Improved resourcing with suitably trained staff willing to focus on psychiatry illness was viewed by many as an essential factor that would improve public and referrer perception and satisfaction with CAMHS. Most respondents (reported by 27) reported a widespread misunderstanding of CAMHS’ “core business”, where the public and, at times referrers, believed it to be a “catch-all service”. Specifically, respondents observed that CAMHS often received inappropriate referrals (e.g., for disability or social care issues), which overburdened CAMHS time and led to bad feeling from those turned away. Respondents called for greater awareness of CAMHS’ status as a specialist service to treat moderate to severe mental illness rather than a non-specialist service for youth with behaviour problems, transient emotional difficulties, or youth with disabilities.

Relatedly, 14 respondents discussed the need to promote realistic expectations from service users regarding the extent and nature of service provision in the context of overstrained resources. Twelve respondents mentioned the need for intersectoral/interagency streamlining. Confusion regarding the division of labour between CAMHS and other agencies, such as disability and social care services, contributed to the unwarranted blaming of CAMHS (n = 6) for failing to provide services outside its statutory remit.

A significant number of respondents believed that the way to correct misinformation and poor perception of CAMHS was by carefully delivered and targeted public campaigns about what CAMHS is and is not: “highlight what CAMHS DOES offer, highlight realistic expectations reservice provision” signposting other services closely related.

Respondents felt a need to be more proactive with a planned rather than reactive media response, focussing on positive and realistic outcomes and advocated “a higher media profile of child psychiatry including radio, television, and articles in newspapers”. Such educational campaigns should be broad, including schools, referrers, and the public, and include family testimonials e.g., “patient feedback where services have gone well”.

Twelve participants observed a general negative bias of attention that in those problematic or deficient aspects of current service provision were more often reported than any beneficial aspects. “There is a lot of emphasis on what’s wrong”, and media focus often in “highlighting negative and sensationalist newspaper articles regarding CAMHS”. Consultants called for increased attention to aspects of service provision that function well, “less scapegoating”, and that the positive experiences of many service users are voiced. Responses from eight consultants implicated the media as a source of CAMHS’ negative reputation and offered the possibility of remediation by the media through the proactive promotion of CAMHS “success stories”. Many consultants perceived that difficulties with access to services (n = 10), general resource deficiencies (n = 6), and staffing deficits (n = 5) contribute to negative perceptions.

### Other comments relating to CAMHS

**Fig. 3:**
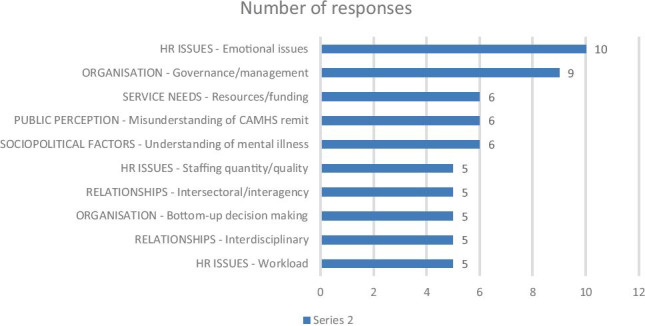
Other comments consultant psychiatrists had relating to CAMHS

The final content analysis was conducted on a general question asking the consultant to offer any other perceptions relating to CAMHS funding, quality, challenges, opportunities, or services in Ireland. Twenty-eight consultants offered responses to this; see Fig. 3.

The most prominent theme emerging from the data was that of the staff’s emotional wellbeing and a need to “acknowledge burn out in CAMHS consultants and consider how to ameliorate same”. The risk of clinician stress and burnout was mentioned by ten respondents, with five expressing concern regarding excessive or inappropriate workload. Respondents used this section to repeat the concerns regarding problems with governance or management (*n* = 9), inadequate resources (*n* = 6), misunderstanding of the CAMHS remit (n = 6), staffing difficulties (n = 5) and salience of interagency (*n* = 5) and interdisciplinary (*n* = 5) relationships.

Six people mentioned a broader socio-cultural factor regarding the public understanding of mental illness. Interestingly, these responses were not generally focused on increasing the public’s mental health awareness. Instead, they emphasised the need to circumscribe CAMHS resources to those with a serious and defined psychiatric disorder rather than young people experiencing general stress or emotional or behavioural difficulties. More appropriate use of specialist services was viewed as essential and beneficial.

Several respondents were concerned with the speciality of child psychiatry “being wiped out” or “dying”, with respondents stating that there were considering or had already opted out of public service to less lucrative but more rewarding private positions, where their ability to manage their workload, team composition, and time was more advantageous. They viewed their “lack of voice in the further development of the CAMHS service” as fundamental to both their feared demise of CAMHS, indicative of “lack of respect for my professionalism” and linked with inefficiencies in service provision.

## Discussion

**Fig. 4:**
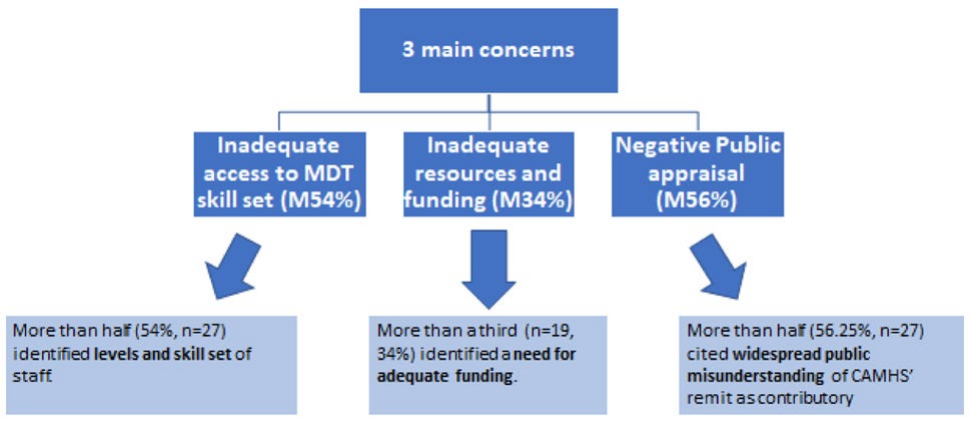
Summary of themes identified in this study & frequency of mentions (M%)

The free-text responses from fifty-two consultant child and adolescent psychiatrists who responded to this survey gave important first-hand insights into CAMHS working conditions. Content analysis indicated that consultants’ perception of CAMHS working conditions revolved around six factors: organisational factors, human resources, adequacy of services, professional relationships, socio-political factors, and public perception. Adequate service provision and allocation of more resources and funding were highlighted by consultants to be closely aligned to work-related stress. Participants believed that not only was additional staffing was needed but there was a pre-requisite skill set required among the MDT staff and a willingness for staff to work flexibly as service demands required (Fig. 4). Consultants also felt that services needed to be evaluated to ensure best use of resources. Consultants reported feeling side-lined and excluded from service planning to the service’s detriment.

There was a strong plea from respondents that the consultant with overall clinical responsibility for their service was more involved in the overall planning, service evaluation, and team complement.

**Fig. 5:**
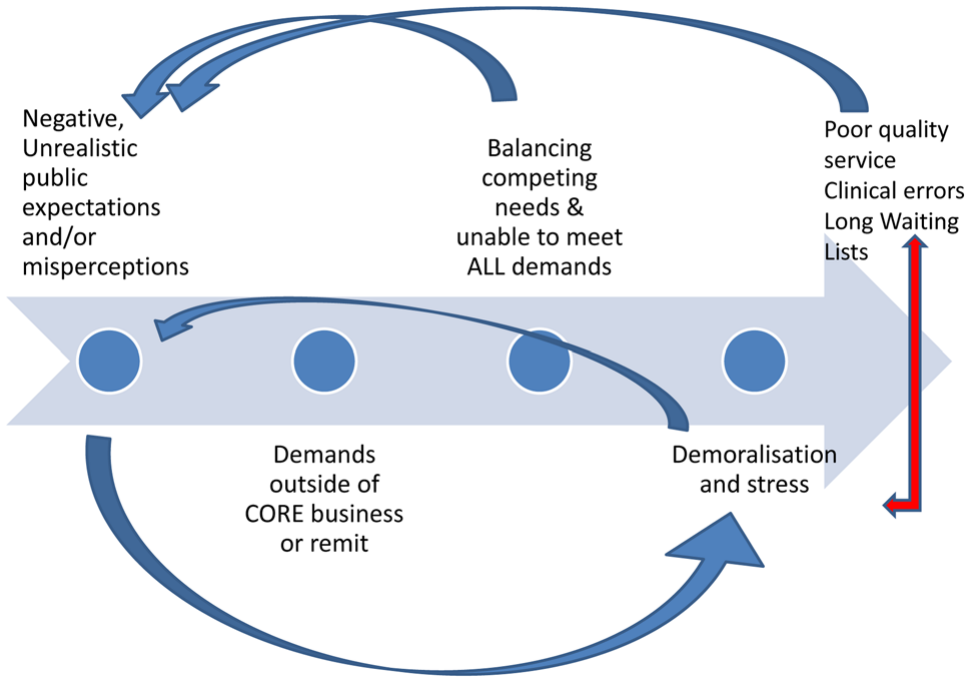
Relationship between public misperceptions and occupational stress

Consultants also identified unrealistic public expectations and misperceptions to be closely associated with occupational burnout. CAMHS’ inability to meet the demands of both referrers and service users, when faced with demands outside of their remit or “core business”, caused demoralisation and stress among staff (Fig. 5). The failure to meet service users’ expectations also engendered negative public perceptions, which further fuelled staff demoralisation. Accepting additional work load would contribute to longer waiting lists, diverting CAMHS resources away from expected and effective treatment of core business, and might not be attainable due to lack of other necessary resources. Furthermore, quality of CAMHS would be reduced by the need to deal with increasing demands and exposed to clinical errors.

**Fig. 6:**
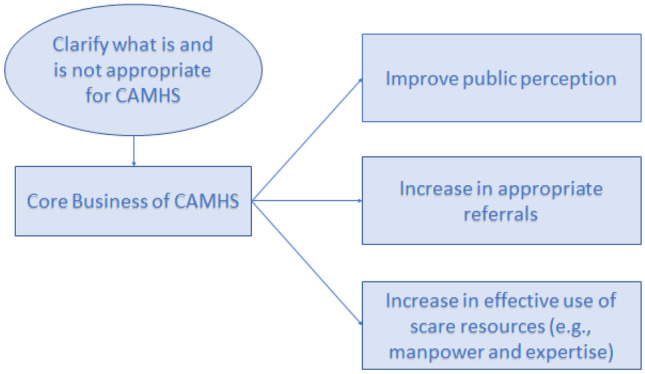
Educational campaign to clarify CAMHS “core business”

Respondents were vocal about the need to engage in educational campaigns which address these misperceptions more actively allowing optimal use of CAMHS and ensuring service users to receive appropriate interventions delivered by staff skilled to provide them (Fig. 6). Consultants were aware of the overlap of needs in families with youth with mental illness, educational difficulties, and social care needs, and requested better interagency joint working.

Levels of occupational stress among consultants in Irish hospital settings [15] and child and adolescent psychiatry settings [10] have already been shown to be high. Data from the quantitative component of this survey reported that 75% of consultants experienced moderate or higher levels of work-related BO, and 72.3% moderate or higher levels of personal BO across community and in-patient settings. Burnout was strongly correlated with a strong consideration to change jobs, present in 69% of the sample. Consultant psychiatrists expressed a lack of confidence in the Irish government’s commitment to CAMHS, with the majority (73%) believing commitment to investment was “never” or “rarely” present and a similar rate (71%) perceiving management by the health authorities to be ineffective and uninformed. Both of these positions were related to consultants experiencing higher levels of burnout [10]. The recently published “Sharing the Vision: A Mental Health Policy for Everyone” [6] offers welcome and considered proposals for improving MH services in Ireland yet fails to address any additional funding or resourcing. This adds little reassurance given the concerns previously raised by almost half of all consultants working in CAMHS. Proceeding to develop new service structures, without adequately addressing the existing deficits, exposes clinicians on the frontline to further occupational stress and burnout.

The COVID-19 pandemic and accompanying mitigation measures have severely impacted the health and wellbeing of the Irish population and economy. Demands placed on healthcare workers have been extraordinary and long-lasting. Evidence from previous pandemics [14] and emerging data from COVID-19 [15] report higher rates of mental illness in both the general population and among clinicians. The extent of the increased demand for CAMHS has more recently been documented [15], with both total numbers and urgent referrals increasing consistently from September 2020. This additional demand places further strain on services already under-resourced and under-funded. In the context where consultants leading the teams were already experiencing high rates of occupational stress, and seriously considering changing jobs, there is an urgent need to carefully examine and modify working conditions in CAMHS. Whilst ensuring adequate staff numbers and skill set are considered vital, addressing the misperceptions by the public as to CAMHS remit offers an additional and low cost method to reduce occupational stress.

### Strengths and limitations

This is the first qualitative exploration of Child and Adolescent Consultant Psychiatrists’ perceptions of factors contributing to levels of occupational stress working in CAMHS. Conducted before COVID-19 pandemic, its findings will be important to consider as clinicians face additional personal and working demands. Although the response rate was lower than hoped, (46%) it was comparable with similar studies [8, 13], and the qualitative nature of the responses allows important insights to be ascertained. The content analysis allowed for some quantification of data, but the methodology would have been further enhanced had more detailed interrogation of the data been possible. Further studies could include individual interviews with a subset of respondents to explore themes identified further.

## Conclusion

This paper explored the personal perspectives of consultant child and adolescent psychiatrists working in CAMHS and identified perceived factors contributing to their levels of occupational stress. Content analysis indicated that consultants’ perception of CAMHS working conditions revolved around six factors: organisational factors, human resources, adequacy of services, professional relationships, socio-political factors, and public perception. The factors identified, coupled with the increased demand for CAMHS services in the aftermath of the COVID-19 pandemic, can exacerbate working conditions and increase occupational stress in professionals working in mental health services. There is an urgent need for engagement and collaboration between consultants leading the service and those responsible for planning and funding. There is an equally urgent need to work with patients, referrers, and the public to ensure adequate understanding of CAMHS’ “core business” and allow delivery of specialist provision to young people with mental illness.

## Data Availability

Requests for reasonable access to study material and data will be considered by contacting corresponding author.

## Appendix I. Thematic analysis codes

**Table:**

Code family	Code	Definition
HR issues	Emotional issues	Emotional effects of job, e.g., stress, morale, frustration, job satisfaction
	Employment conditions	Working conditions, e.g., leave, disciplinary procedures, salary, role definition
	Staffing quantity/quality	Appropriate staff numbers or expertise. Include reference to recruitment/retention
	CPD	Training or professional development of staff. Include reference to performance reviews
	Workload	Quantity of work or specific responsibilities
Organisational factors	Bottom-up decision making	More input from clinical staff into operational/policy decisions
	Standardised clinical procedures	Implementation of consistent admissions and therapeutic pathways. Include reference to evidence-based practice
	Governance/management	Critical comments on CAMHS governance, management, or leadership. Include reference to planning and strategy
	Organisational structure	Criticisms of or recommended changes to organisational structure (e.g., location or composition of services)
Public perception	Blame/accountability	CAMHS/clinicians held accountable for factors outside their responsibilities
	Misunderstanding of CAMHS remit	Misunderstanding from the public, government, or other agencies regarding CAMHS role (specialist medical service, not generic emotional support) and populations served (incl. reference to unclear referral policies)
	Focus on positives	Overemphasis on flaws/failings — need more focus on achievements and positive patient experiences
	Realistic expectations	Need for service users to have realistic expectations of service provision and awareness of resource constraints
	Media	Reference to the media’s role in promoting awareness/misconception of CAMHS
Relationships	Primary care	Importance of primary care and its resourcing
	Interdisciplinary	Importance of effective interdisciplinary work
	Intersectoral/interagency	Importance of cooperation with other aspects of health, social care and education system, and clarity redistribution of responsibilities
	Interpersonal	Importance of relationships with colleagues
	AMHS	Importance of consistency with adult services
Service needs	Access	Problems with access to services. Include reference to long waiting lists and on-call/24-h services
	Administrative/IT support	Need for improved administrative or IT resources. Includes reference to medical record systems
	Evaluation	Evaluation/audit of service performance and outcomes
	Inpatient services	More resources for inpatient services (e.g., beds)
	Outpatient services	More resources for outpatient services (e.g., day hospitals)
	Physical resources	Need for better physical accommodation of services (e.g., office space)
	Specific interventions	Stated need for investment in specific interventions (e.g., certain therapies)
	Resources/funding	Need for greater funding or generic ‘resources’
Sociopolitical factors	Understanding of mental illness	Need for better understanding/more precise definition of mental illness
	Economic factors	Reference to macro-economic policy
	Political/policy factors	Reference to political factors or government policy affecting CAMHS. Includes reference to Vision for Change
	Cultural values	Influence of cultural values, e.g., regarding children's rights
	Geography	Reference to the regional distribution of staff/services
